# The Effect of An Angiogenic Cytokine on Orthodontically Induced Inflammatory Root Resorption 

**DOI:** 10.22074/cellj.2016.4323

**Published:** 2016-05-30

**Authors:** Massoud Seifi, Ali Lotfi, Mohammad Reza Badiee, Zahra Abdolazimi, Parisa Amdjadi, Majid Bargrizan

**Affiliations:** 1 Department of Orthodontics, Dental School, Shahid Beheshti University of Medical Sciences, Tehran, Iran; 2 Department of Oral and Maxillofacial Pathology, Dental School, Shahid Beheshti University of Medical Sciences, Tehran, Iran; 3 Dentofacial Deformities Research Center, Research Institute of Dental Sciences, Shahid Beheshti University of Medical Science, Tehran, Iran; 4 Department of Pedodontics, Dental School, Shahid Beheshti University of Medical Sciences, Tehran, Iran; 5 Department of Dental Materials, Dental School, Shahid Beheshti University of Medical Sciences, Tehran, Iran

**Keywords:** Root Resorption, Basic Fibroblast Growth Factor, Angiogenesis, Rat

## Abstract

**Objective:**

Orthodontically induced inflammatory root resorption (OIIRR) is an undesirable sequel of tooth movement after sterile necrosis that takes place in periodontal ligament due to blockage of blood vessels following exertion of orthodontic force. This study sought to assess the effect of an angiogenic cytokine on OIIRR in rat model.

**Materials and Methods:**

In this experimental animal study, 50 rats were randomly divided into 5 groups of 10 each: E10, E100 and E1000 receiving an injection of 10, 100 and 1000 ng of basic fibroblast growth factor (bFGF), respectively, positive control group (CP) receiving an orthodontic appliance and injection of phosphate buffered saline (PBS) and the negative control group (CN) receiving only the anesthetic agent. A nickel titanium coil spring was placed between the first molar and the incisor on the right side of maxilla. Twenty-one days later, the rats were sacrificed. Histopathological sections were made to assess the number and area of resorption lacunae, number of blood vessels, osteoclasts and Howship’s lacunae. Data were statistically analyzed using ANOVA and Tukey’s honest significant difference (HSD) test.

**Results:**

Number of resorption lacunae and area of resorption lacunae in E1000 (0.97 ± 0.80 and 1. 27 ± 0.01×10^-3^, respectively) were significantly lower than in CP (4.17 ± 0.90 and 2.77 ± 0.01×10-3, respectively, P=0.000). Number of blood vessels, osteoclasts and Howship’s lacunae were significantly higher in E1000 compared to CP (P<0.05).

**Conclusion:**

Tooth movement as the outcome of bone remodeling is concomitant with the formation of sterile necrosis in the periodontal ligament following blocked blood supply. Thus, bFGF can significantly decrease the risk of root resorption by providing more oxygen and angiogenesis.

## Introduction

Root resorption is defined as the loss of cementum and dentin that is considered as a physiological or a pathological phenomenon. It is classified into two groups of internal and external root resorption. External root resorption is among the unwanted side effects resulting from orthodontic treatment (1). Root resorption due to orthodontic treatment seems to be a multifactorial phenomenon depending on factors such as the amount and type of applied orthodontic forces, duration of orthodontic treatment, preexisting condition of root resorption, root morphology, biological and genetic factors, ethnicity and patient’s health status (1,2). Some studies have reported the prevalence of 93% for root resorption in adolescents under orthodontic treatment. Moderate to severe apical root resorption (>2mm to <1/3 of the root length) occurs in 12-17% and severe apical root resorption (more than 1/3 of the root length) in 1-5% of patients under orthodontic therapy (3,4). 

Following the application of orthodontic force, pressure areas develop in the periodontal ligament (PDL). In pressure areas, PDL is compressed in a confined area resulting in subsequent blocked blood supply and deranged cell differentiation which further leads to cell degeneration and consequent formation of a glasslike hyalinized region. Hyalinization is an inevitable process during orthodontic treatment. Since cells cannot differentiate into osteoclasts in the hyalinized area, tooth movement enters the lag phase which lasts up until the mentioned area is eliminated. Invasion of cells and blood vessels from the intact neighboring periodontal ligament results in elimination of this area (1,3). Recent studies have demonstrated that giant cells belonging to the mononuclear phagocyte system remove the necrotic tissue and also eliminate part of the adjacent root cementum resulting in unwanted root resorption (5). Furthermore, it has been shown that longer treatment times increase the risk of root resorption (1,2,6). 

At present, there is a growing interest for the use of biologic molecules such as the prostaglandins (PGs), vascular endothelial growth factor (VEGF) and basic fibroblast growth factor (bFGF) to accelerate orthodontic tooth movement. Noda et al. (7) used platelet-derived growth factor (PDGF) to treat root resorption after extraction and reimplantation of a partially denuded tooth. Their results showed that new periodontal ligament tissue was formed, while occurrence and extent of root resorption decreased. Shiratani et al. (8) showed that use of FGF-2 reduced the occurrence of root resorption following delayed autotransplantation of tooth. FGF-2 is a cytokine from the large family of FGF. Role of this molecule is similar to that of VEGF and plays a part in migration and proliferation of endothelial cells, angiogenesis under *in vivo* conditions and bone remodeling (9,10). This growth factor also improves vascularization, wound healing, control and formation of bone mass and proliferation of osteoclasts (11). 

One of the important goals of orthodontic therapy is to reduce its unwanted side effects. The long-term treatment course not only compromises the patient’s oral health status that leads to subsequent development of caries, but is also considered as a risk factor for development of root resorption. If angiogenesis is increased by the use of bFGF at the predetermined site, we may be able to prevent the formation of sterile necrosis and shorten the subsequent lag phase in orthodontic treatment course. By doing so, it is possible to facilitate orthodontic tooth movement and decrease root resorption. The present study aimed at determining the effect of various doses of bFGF on orthodontically induced inflammatory root resorption (OIIRR) in rat model. 

## Materials and Methods

In this experimental animal study, approved protocols (Institutional Review Board) regarding animal studies were precisely followed. Ethical approval was obtained from the Ethics Committee of Shahid Beheshti University of Medical Science. A total of 50 male Wistar rats with a mean age of 4 months and a mean weight of 330 ± 30 g were held under equive alent situation of light and nutrition for two weeks to become familiar with the new environment. During this time period and throughout the study, soft diet (grinded food) was provided for all groups under study. Drinking water of animals was also replaced with distilled water. Rats were randomly divided into 5 groups of 10 each using simple random sampling method. They were color-coded and placed in separate coded cages. At baseline, each rat was weighed using a digital scale (Shimadzu, Japan) and prepared for anesthesia induction. Anesthesia was induced with 120 mg/kg Ketamine (Ketamine hydrochloride, Rotexmedica, Germany) and 5 mg/kg Xylazine (Bayer AG, Germany) that were administered intraperitoneally using an insulin syringe. 

**Fig.1:**
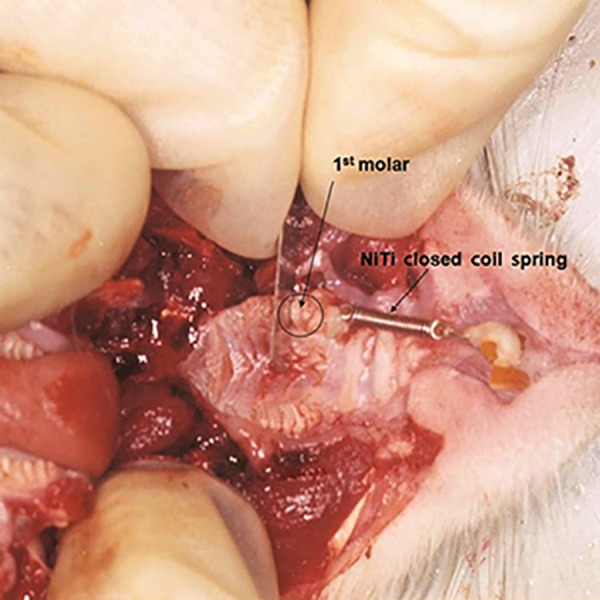
Placement of orthodontic appliance.

Right maxillary first molar was attached to the central incisor of the same side using an orthodontics NiTi closed coil (0.010×0.030 inch, 3M Unitek, USA) with an eyelet (9 mm) and a stainless steel ligature wire (0.01 inch) that was fixed with self-cure composite resin (3M Unitek, USA) (Fig.1). The applied force was 60 g measured by the force-meter. The type of tooth movement was tipping. 

Phosphate buffered saline (PBS) was used to dilute the bFGF (12,14). Since the traumatic injuries resulting from injection may affect the results of study, ten rats in the positive control group (CP) received an injection of 0.02 cc PBS and an orthodontic appliance. The negative control group (CN) did not receive any orthodontic appliance or injection of growth factor. This group only received the anesthetic agent during the study period. The remaining 30 rats were randomly divided into three groups of E10, E100 and E1000. Rats in the E10 group received an injection of 0.02 cc of 10 ng bFGF (Royan Institute, Iran). Rats in the E100 group received an injection of 0.02 cc of 100 ng bFGF and rats in the E1000 group received an injection of 0.02 cc of 1000 ng bFGF. Selected doses of bFGF were injected into the buccal vestibular mucosa adjacent to the mesial root of the first molar tooth. 

After 21 days, rats were sacrificed by saturated chloroform inhalation. Since the cells cannot differentiate in hyalinized area during the application of forces, 21 days is an appropriate time for cell differentiation (15). Feeler gauge was used to proof that orthodontic tooth movement had happened (Fig.1). 

**Fig.2:**
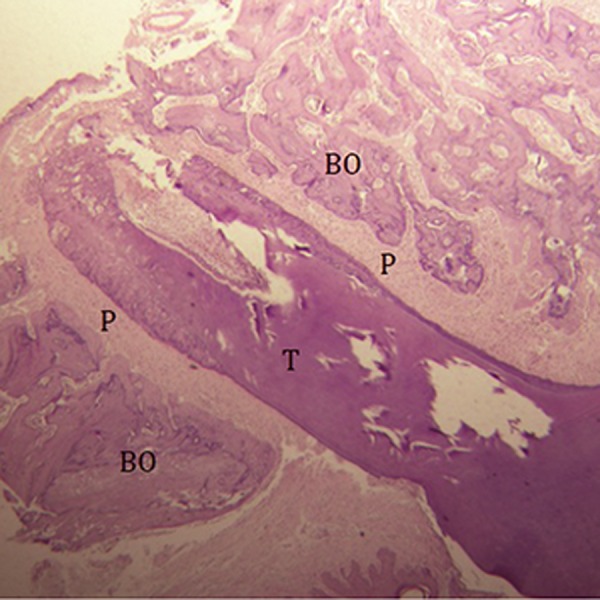
Longitudinal section (×4) of the mesial root of the right maxillary first molar. BO; Alveolar bone, P; Periodontal ligament and T; Tooth.

**Fig.3:**
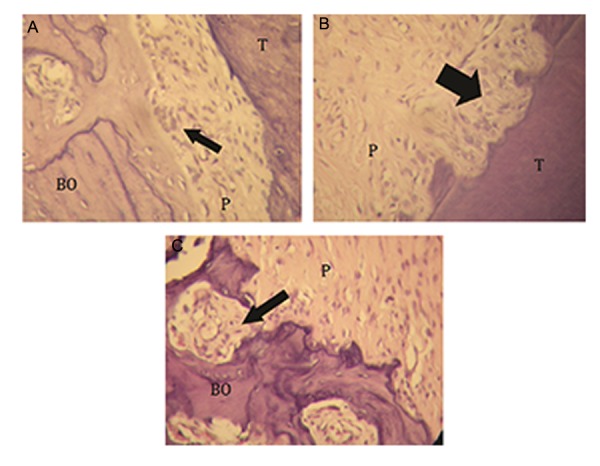
A. Osteoclast-like cells (arrow), B. Resorption lacunae (arrow) and C. Howship’s lacunae (arrow) observed at the mesial surface of the mesial root of right maxillary first molar (magnification: ×40). BO; Alveolar bone, P; Periodontal ligament and T; Tooth.

**Fig.4:**
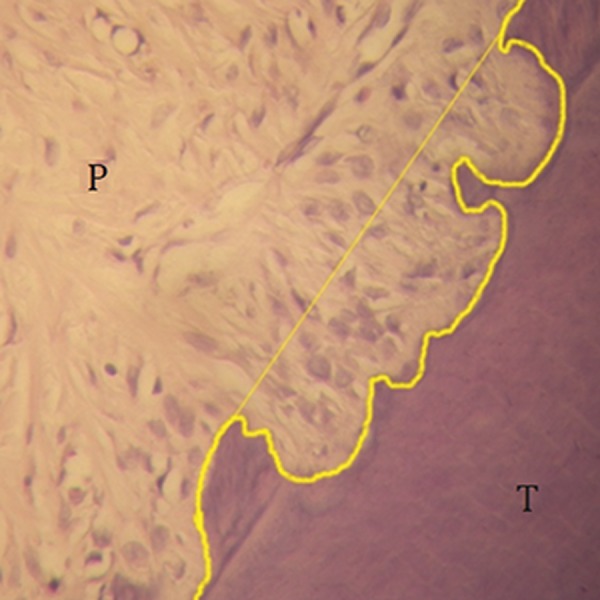
Calculation of the area of resorption lacunae. P; Periodontal ligament and T; Tooth.

## Statistical analysis 

Obtained data were analyzed using the SPSS (SPSS Inc., USA) version 17.0, ANOVA, Tukey’s honest significant difference (HSD) and t tests. A P value less than 0.05 was considered statistically significant. 

## Results

**Fig.5:**
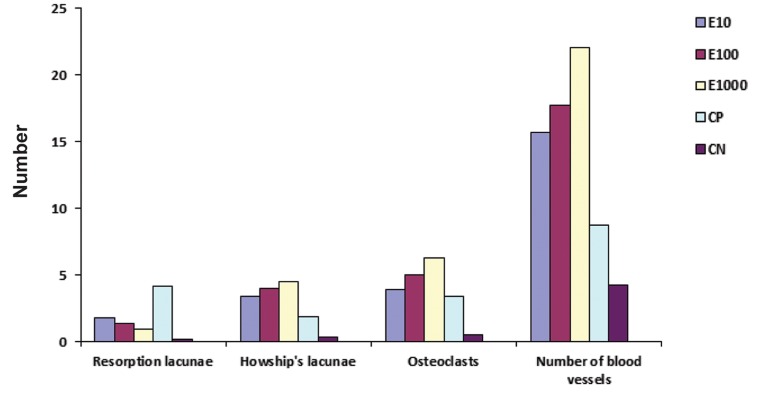
Number of resorption lacunae, Howship’s lacunae, osteoclasts and blood vessels cross sections in different groups.

**Fig.6:**
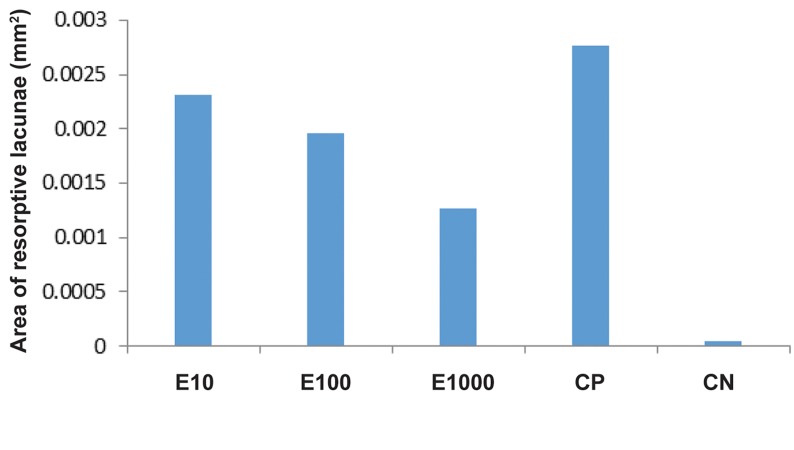
Area of resorption lacunae in different groups. bFGF; Basic fibroblast growth factor, E10; Test group receiving 0.02 cc of 10 ng bFGF, E100; Test group receiving 0.02 cc of 100 ng bFGF, E1000; Test group receiving 0.02 cc of 1000 ng bFGF, CP; Positive control group receiving 0.02 cc of phosphate buffered saline, and CN; Negative control group without the orthodontic appliance.

For the assessment of root resorption, the mean number and area of resorption lacunae were calculated (Table 1). ANOVA revealed a statistically significant difference in number of resorption lacunae at the mesial surface (P<0.05, F=41.061). E10 and E100 groups showed a statistically significant difference in the number of resorption lacunae (P<0.05) as compared with the CP and CN groups. Number of resorption lacunae in the E1000 group was significantly lower than that in the CP group (P<0.05); however, there was no statistically significant difference as compared with the related values of E10, E100 and CN groups (Fig.5). The greatest area of resorption lacunae was observed in the CP group (0.277 ± 0.001×10^-2^ mm^2^, Table 1). Area of resorption lacunae in E1000 was significantly less than that in E10 and CP and more than that in CN group. E10 and E100 had no statistically significant difference with CP in this respect (Fig.6). Another variable under study was the number of Howship’s lacunae (Table 1). The difference in number of Howship’s lacunae was statistically significant (P<0.05) among different groups, indicating that the values in the test groups were significantly higher than that in controls (P<0.05). Among the test groups according to Tukey’s HSD test, the mean number of Howship’s lacunae in E1000 was significantly higher than that in E10 group (P<0.05); however, no such difference was detected between the E10 and E100 or E100 and E1000 groups (Fig.6). Number of osteoclasts was also evaluated (Table 1). Significant differences were also found in number of osteoclasts among different groups (P<0.05). This rate was significantly higher in the test groups (E10, E100, and E1000) compared to the controls. Among the test groups, the mean number of osteoclast cells in the E1000 group was significantly higher than that in E10 group. This rate in E100 was significantly higher than that in E10 as well (Fig.6). The greatest number of blood vessels was found in E1000 group (22.0666 ± 1.6239, Table 1). ANOVA found significant differe ences in number of blood vessels in different groups (P<0.05). Tukey’s HSD test was used for pairwise (inter-group) comparison of groups, indicating that number of blood vessels was significantly different among groups (Fig.6). 

**Table 1:** The mean values obtained for variables in different groups


Groups	E10	E100	E1000	CP	CN

Number of resorptive lacunae	1.8000 ± 0.7235	1.3333 ± 0.7653	0.9667 ± 0.7986	4.1667 ± 0.8923^*^	0.2133 ± 0.4172
Area of resorptive lacunae (mm 2 )	0.231 ± 0.016×10^-2**^	0.196 ± 0.001×10^-2^	0.127 ± 0.001×10^-2^	0.277 ± 0.001×10^-2**^	0.004 ± 0.001×10^-2**^
Number of vessel cross sections	15.7667 ± 1.2277^**^	17.7333 ± 0.9401^**^	22.0666 ± 1.6239	8.7000 ± 1.4611^**^	4.9333 ± 1.4555^**^
Number of Howship’s lacunae	3.4000 ± 0.9660^**^	4.0667 ± 0.8577	4.5000 ± 0.5931	1.9333 ± 0.4661^*^	0.3000 ± 0.2459^*^
Number of osteoclasts	3.9667 ± 0.4830^†^	5.0333 ± 0.7927	6.3333 ± 0.6666	3.4000 ± 0.9660^*^	0.5000 ± 0.1756^*^


bFGF; Basic fibroblast growth factor, E10; Test group receiving 0.02 cc of 10 ng bFGF, E100; Test group receiving 0.02 cc of 100 ng bFGF, E1000; Test group receiving 0.02 cc of 1000 ng bFGF, CP; Positive control group receiving 0.02 cc of phosphate buffered saline, CN; Negative control group without the orthodontic appliance, *; Statistically significant difference with E10, E100, and E1000 groups, **; Statistically significant difference with E1000 and †; Statistically significant difference with E100 and E1000.

## Discussion

FGF-2 is known as an important cytokine belonging to the FGF family. This growth factor has multiple effects on cells and tissues such as induction of endothelial cell proliferation, angiogenesis, tissue remodeling and wound healing, proliferation and differentiation of osteoblasts, osteoclasts and macrophage chemotaxis (16,19). 

Miyagawa et al. (20) reported that expression of VEGF increased in the PDL cells adjacent to hyalinized tissue and alveolar bone on the compressive side and plays an important role in angiogenesis. However, other angiogenic factors such as FGF-2, tumor-necrosis factor-α (TNF-α) and transforming growth factor-β (TGF-β) may also play a role in this regard. Kohno et al. (15) evaluated the effect of injection of recombinant human VEGF (rhVEGF) on tooth movement in rats. They showed that no significant differences existed between the test and control groups up until day 15. However, the difference between the two groups became statistically significant at days 18-21. They attributed this difference to the lack of osteoclastic induction and subsequent root resorption in rhVEGF group up until day 14. In another study conducted by the same researchers, they concluded that injection of anti-VEGF polyclonal antibody in rats during tooth movement results in a significant decrease in number of osteoclasts, rate of tooth movement and rate of relapse (21). Lu-lu et al. (22) reported that injected bFGF (extrinsic) plays a key role in PDL remodeling during orthodontic tooth movements. Angiogenic and osteogenic properties of bFGF as well as its ability for induction of fibroblasts and macrophages have been demonstrated in several *in vivo* and *in vitro* studies (15,18,23). 

Blocked blood supply and reduced oxygenation at the pressure side and subsequent formation of sterile necrosis are among the causes of decelerated tooth movement following the primary phase. Application of bFGF is expected to eliminate the lag phase of tooth movement by increase of angiogenesis and subsequent oxygenation and induction of macrophages and secondary messengers involved in tooth movements. On the other hand, the potential of different cell types i.e. osteoblasts and osteoclasts for proliferation and differentiation and their critical role in PDL remodeling during orthodontic tooth movement also contribute to this process (10,19). 

Parameters evaluated for root resorption in our study were almost similar to those of other studies (24,27). Akin et al. (24) in a study assessed the number of resorption lacunae, osteoclasts and capillary vascularization for evaluation of root resorption in rats. Kale et al. (25) studied the number of Howship’s lacunae, capillaries and osteoblasts in prepared samples. Seifi et al. (26) used computer software to assess the area of resorption lacunae in mesial root of first molar in rat model. In another study by Seifi and Aghaeei Pour (27), they used Adobe Photoshop Cs3 to assess root resorption. 

In our study, although the number of resorption lacunae in the E1000 group was significantly lower than that in the CP group, no significant differences were detected between E1000 with E10, E100 or CN in this respect. It should be mentioned that the mean depth of resorption lacunae was the lowest in the CN group. However, this rate was not equal to zero. This finding confirms that root resorption can also occur in teeth that have never undergone orthodontic treatment (28). The anesthetic agent may play a part in this regard as well. 

Another variable evaluated for root resorption was the mean area of resorption lacunae which in the E1000 group was significantly lower than that in the E10 and CP groups and significantly higher than that in the CN group. The results of studies by Kurol and Owman-Moll (3), Brudvik and Rygh (29), as well as Brenzniak and Wasserstein (5) indicated that root resorption at areas adjacent to or beneath the sterile necrosis occurs during the removal of necrotic tissue by the macrophages. Lower number of resorption lacunae in the groups receiving an injection of bFGF can be due to the effect of this material on reducing the size or duration of sterile necrosis area that subsequently decreases adjacent root resorption. Number of resorption lacunae in the E1000 group was different from that in the CN group (without orthodontic tooth movement), but this difference was not statistically significant. This finding indicates that injection of a higher dose of bFGF (1000 ng) can decrease the number of resorption lacunae to the original level as in a tooth with no orthodontic movement. On the other hand, although in terms of number of resorption lacunae, no significant differences existed between the E10 and E1000 groups, the mean area of resorption lacunae in the E1000 group was significantly lower than that in the E10 group. It indicates that smaller resorption lacunae are present in the E1000 group, while higher doses of this growth factor are capable of better protection of root surfaces against resorption following orthodontic movement. On the other hand, it has been demonstrated that chemotactic factors; extracellular matrix; adhesion molecules; and growth factors such as insulin growth factor-1 (IGF-I), FGFs, epidermal growth factor (EGF), bone morphogenetic proteins (BMPs) and TGF-β present in cementum matrix can result in influx and differentiation of pre-cementoblast cells that per se play a significant role in repair of root resorption (30,36). This finding also justifies the reduced root resorption in the groups receiving growth factor compared to the controls. 

Number of Howship’s lacunae and osteoclasts are considered as indicative factors of bone remodeling in an area. Number of resorption lacunae was significantly higher in the groups receiving bFGF cytokine compared to the controls. Among the test groups, the mean number of Howship’s lacuna in the E1000 group was significantly higher than that in the E10 group. 

Osteoclasts are multinuclear cells that are greatly involved in bone resorption around the teeth. Significant differences existed between the groups receiving bFGF cytokine and the control groups. Lin et al. (37) stated that FGF-8 plays a key role in bone metabolism and remodeling through the uncoupling of osteoblastic and osteoclastic activities. Manabe et al. (38) reported that osteoclastogenesis following an increase in endogenous FGF-2 level in the synovial fluid plays a role in joint destruction in rheumatoid arthritis (RA) patients. Greater number of Howship’s lacuna and osteoclasts in the groups receiving growth factor compared to the control groups indicates induced chemotaxis of osteoclasts and osteogenesis by this cytokine. Considering the higher rate of mentioned parameters in the E1000 group (that had received higher dosage of growth factor) compared to other test groups, we can state that the effect of bFGF is dose-dependent. Kaku et al. (39) evaluated the effect of rhVEGF on osteoclast induction. Their results revealed that the highest numbers of osteoclasts are obtained following injection of minimum of 0.5 µg rh-VEGF. Elimination of hyalinized tissue requires the formation of new blood vessels (angiogenesis) and removal of necrotic tissue. Thus, number of blood vessels was evaluated as well. The number of vessels in the E1000 group was significantly higher than that in the other groups. Chang et al. (40) examined the effect of endothelial cell growth factor (ECGF) on cell migration patterns during the orthopedic expansion and concluded that the angiogenic growth factor can increase preosteoblast population that results in new bone formation and improves treatment stability. 

Our study results were in accordance with those of Chang et al. Both studies demonstrated that by the application of growth factors, number of preosteoclasts and Howship’s lacunae increase. In a study by Jansen et al. (41), influx of cells and increased rate of vascularization by recombinant FGF-2-loaded collagen scaffolds produced in *Escherichia coli (E. coli)* in the oral mucosa of rats were noted. FGF-2 caused faster influx of host cells and subsequently faster disappearance of the scaffolds, an increase in the rate of vascularization, improvement of grafting, a decrease in the myofibroblast score and consequently reduction of contraction during the process of repair. Behr et al. (42) demonstrated that FGF-9 has a critical role in proliferation of pre-osteoblasts, osteogenesis and angiogenesis in long-term bone repair. In a study by Parsons-Wingerter et al. (43), it was shown that application of FGF-2 compared to PBS could considerably increase the density of small vessels. Willems et al. (18) evaluated the longterm effect of microspheres containing 10.5 µg VEGF, bFGF and VEGF+bFGF on the angiogenesis and osteogenesis in an allograft transplanted in a rat model. Angiogenesis and bone remodeling at 4 and 18 weeks were significantly higher in the VEGF group compared to the controls. No considerable angiogenic or osteogenic response was observed in the bFGF group. No synergistic effect was also noted for FGF-2+VEGF. They reported this response was due to the presence of a limited functional threshold for bFGF. Over-expression and long-term application of bFGF can lead to apoptosis of cells or osteoblasts. Therefore, range of function of bFGF is highly dose-dependent (17). Sun et al. (44) also showed that the angiogenic effects of bFGF and VEGF are dose-dependent (1040 ng/mL) and a synergy exists between these two. It means that the results of Willems et al. (18) are contrary to our findings. This difference can be attributed to the longer duration of their study and use of a higher dose of bFGF in the form of microsphere that results in continuous and longer release of this growth factor. Duration of the present experiment was 21 days. It is require to maintain this time period for osteoclast induction according to study by Kohno et al. (15). Osteoblast development seems to be more sensitive to changes in dosage of bFGF compared to other factors such as VEGF since long-term exposure to this growth factor causes apoptosis of mature osteoblasts; therefore, this growth factor has a limited range of therapeutic effects (45,47). 

## Conclusion

Injection of bFGF resulted in a significant increase in rate of angiogenesis, number of pre-osteoclasts and number of Howship’s lacunae and a reduction in number and area of resorption lacunae on the root surface. The effect of bFGF was dosedependent and its greatest effect was inserted when 1000 ng of bFGF was administered in rat model. Results of the present study indicate that local application of angiogenic factors such as bFGF can decrease the duration of orthodontic treatment and prevalence of related complications i.e. root resorption. However, further studies are required on this subject. 

## References

[1] Ramanathan C, Hofman Z (2006). Root resorption in relation to orthodontic tooth movement. Acta Medica (Hradec Kralove).

[2] Weltman B, Vig KW, Fields HW, Shanker S, Kaizar EE (2010). Root resorption associated with orthodontic tooth movement: a systematic review. Am J Orthod Dentofacial Orthop.

[3] Kurol J, Owman-Moll P (1998). Hyalinization and root resorption during early orthodontic tooth movement in adolescents. Angle Orthod.

[4] OwmanMoll P, Kurol J (2000). Root resorption after orthodontic treatment in highand low-risk patients: analysis of allergy as a possible predisposing factor. Eur J Orthod.

[5] Brezniak N, Wasserstein A (2002). Orthodontically induced inflammatory root resorption.Part I: the basic science aspects. Angle Orthod.

[6] Fox N (2005). Longer orthodontic treatment may result in greater external apical root resorption. Evid Based Dent.

[7] Noda K, Seshima F, Okubo N, Ishii Y, Ota M, Yamada S (2012). Effect of platelet-derived growth factor-BB on root resorption after reimplantation of partially denuded tooth in dog. Dent Traumatol.

[8] Shiratani S, Ota M, Fujita T, Seshima F, Yamada S, Saito A (2012). Effect of basic fibroblast growth factor on root resorption after delayed autotransplantation of tooth in dogs. Oral Surg Oral Med Oral Pathol Oral Radiol.

[9] Qu D, Li J, Li Y, Gao Y, Zuo Y, Hsu Y (2011). Angiogenesis and osteogenesis enhanced by bFGF ex vivo gene therapy for bone tissue engineering in reconstruction of calvarial defects. J Biomed Mater Res A.

[10] Derringer KA, Linden RW (2004). Vascular endothelial growth factor, fibroblast growth factor 2, platelet derived growth factor and transforming growth factor beta released in human dental pulp following orthodontic force. Arch Oral Biol.

[11] Sako E, Hosomichi J (2010). Alteration of bFGF expression with growth and age in rat molar periodontal ligament. Angle Orthod.

[12] Hakami Z, Kitaura H, Kimura K, Ishida M, Sugisawa H, Ida H (2015). Effect of interleukin-4 on orthodontic tooth movement and associated root resorption. Eur J Orthod.

[13] Fujimura Y, Kitaura H, Yoshimatsu M, Eguchi T, Kohara H, Morita Y (2009). Influence of bisphosphonates on orthodontic tooth movement in mice. Eur J Orthod.

[14] Hashimoto F, Kobayashi Y, Mataki S, Kobayashi K, Kato Y, Sakai H (2001). Administration of osteocalcin accelerates orthodontic tooth movement induced by a closed coil spring in rats. Eur J Orthod.

[15] Kohno S, Kaku M, Tsutsui K, Motokawa M, Ohtani J, Tenjo K (2003). Expression of vascular endothelial growth factor and the effects on bone remodeling during experimental tooth movement. J Dent Res.

[16] Depprich RA, Meyer U, Meyer T, Handschel J, Wiesmann HP (2009). Biomolecule use in tissue engineering. Fundamentals of tissue engineering and regenerative medicine.

[17] Okada-Ban M, Thiery JP, Jouanneau J (2000). Fibroblast growth factor-2. Int J Biochem Cell Biol.

[18] Willems WF, Larsen M, Friedrich PF, Shogren KL, Bishop AT (2012). Induction of angiogenesis and osteogenesis in surgically revascularized frozen bone allografts by sustained delivery of FGF-2 and VEGF. J Orthop Res.

[19] Feito MJ, Lozano RM, Alcaide M, Ramírez-Santillán C, Arcos D, Vallet-Regí M (2011). Immobilization and bioactivity evaluation of FGF-1 and FGF-2 on powdered silicondoped hydroxyapatite and their scaffolds for bone tissue engineering. J Mater Sci Mater Med.

[20] Miyagawa A, Chiba M, Hayashi H, Igarashi K (2009). Compressive force induces VEGF production in periodontal tissues. J Dent Res.

[21] Kohno S, Kaku M, Kawata T, Fujita T, Tsutsui K, Ohtani J (2005). Neutralizing effects of an anti-vascular endothelial growth factor antibody on tooth movement. Angle Orthod.

[22] Lu-lu Q, Ying Z, Peng-jun L, Jian-Hua F, Shu-yan L (2009). Effect of recombinant mouse fibroblast growth factor-basic on tooth movement during orthodontic treatment in rats with periodontitis. Acta Academiae Medicinae CPAF.

[23] Montesano R, Vassalli JD, Baird A, Guillemin R, Orci L (1986). Basic fibroblast growth factor induces angiogenesis in vitro. Proc Natl Acad Sci USA.

[24] Akin E, Gurton AU, Olmez H (2004). Effects of nitric oxide in orthodontic tooth movement in rats. Am J Orthod Dentofacial Orthop.

[25] Kale S, Kocadereli I, Atilla P, Aşan E (2004). Comparison of the effects of 1,25 dihydroxycholecalciferol andprostaglandin E2 on orthodontic tooth movement. Am J Orthod Dentofacial Orthop.

[26] Seifi M, Eslami B, Saffar AS (2003). The effect of prostaglandin E2 and calcium gluconate on orthodontic tooth movement and root resorption in rats. Eur J Orthod.

[27] Seifi M, Aghaeei Pour N (2009). Effect of Pamidronate on tooth movement and root resorption in rat. Shahid Beheshti Univ Dent J.

[28] Lopatiene K, Dumbravaite A (2008). Risk factors of root resorption after orthodontic treatment. Stomatologija.

[29] Brudvik P, Rygh P (1994). Root resorption beneath the main hyalinized zone. Eur J Orthod.

[30] Grzesik WJ, Narayanan AS (2002). Cementum and periodontal wound healing andregeneration. Crit Rev Oral Biol Med.

[31] Bosshardt DD, Schroeder HE (1996). Cementogenesis reviewed: a comparison between human premolars and rodent molars. Anat Rec.

[32] Götz W, Lossdörfer S, Krüger U, Braumann B, Jäger A (2003). Immunohistochemical localization of insulin-like growth factor-II and its binding protein-6 in human epithelial cells of Malassez. Eur J Oral Sci.

[33] Götz W, Heinen M, Lossdörfer S, Jäger A (2006). Immunohistochemical localization of components of the insulin-like growth factor system in human permanent teeth. Arch Oral Biol.

[34] Götz W, Kunert D, Zhang D, Kawarizadeh A, Lossdörfer S, Jäger A (2006). Insulin-like growth factor system components in the periodontium during tooth root resorption and early repair processes in the rat. Eur J Oral Sci.

[35] Bosshardt DD (2005). Are cementoblasts a subpopulation of osteoblasts or a unique phenotype?. J Dent Res.

[36] Jäger A, Kunert D, Friesen T, Zhang D, Lossdörfer S, Götz W (2008). Cellular and extracellular factors in early root resorption repair in the rat. Eur J Orthod.

[37] Lin JM, Callon KE, Lin JS, Watson M, Empson V, Tong PC (2009). Actions of fibroblast growth factor-8 in bone cells in vitro. Am J Physiol Endocrinol Metab.

[38] Manabe N, Oda H, Nakamura K, Kuga Y, Uchida S, Kawaguchi H (1999). Involvement of fibroblast growth factor-2 in joint destruction of rheumatoid arthritis patients. Rheumatology (Oxford).

[39] Kaku M, Kohno S, Kawata T, Fujita I, Tokimasa C, Tsutsui K (2001). Effects of vascular endothelial growth factor on osteoclast induction during tooth movement in mice. J Dent Res.

[40] Chang HN, Garetto LP, Katona TR, Potter RH, Roberts WE (1996). Angiogenic induction and cell migration in an orthopaedically expanded maxillary suture in the rat. Arch Oral Biol.

[41] Jansen RG, van Kuppevelt TH, Daamen WF, KuijpersJagtman AM, Von den Hoff JW (2009). FGF-2-loaded collagen scaffolds attract cells and blood vessels in rat oral mucosa. J Oral Pathol Med.

[42] Behr B, Leucht P, Longaker MT, Quarto N (2010). Fgf-9 is required for angiogenesis and osteogenesis in long bone repair. Proc Natl Acad Sci USA.

[43] Parsons-Wingerter P, Elliott KE, Clark JI, Farr AG (2000). Fibroblast growth factor-2 selectively stimulates angiogenesis of small vessels in arterial tree. Arterioscler Thromb Vasc Biol.

[44] Sun XT, Ding YT, Yan XG, Wu LY, Li Q, Cheng N (2004). Angiogenic synergistic effect of basic fibroblast growth factor and vascular endothelial growth factor in an in vitro quantitative microcarrier-based three-dimensional fibrin angiogenesis system. World J Gastroenterol.

[45] Mayr-Wohlfart U, Waltenberger J, Hausser H, Kessler S, Günther KP, Dehio C (2002). Vascular endothelial growth factor stimulates chemotactic migration of primary human osteoblasts. Bone.

[46] Marie PJ (2003). Fibroblast growth factor signaling controlling osteoblast differentiation. Gene.

[47] Mansukhani A, Bellosta P, Sahni M, Basilico C (2000). Signaling by fibroblast growth factors (FGF) and fibroblast growth factor receptor 2 (FGFR2)-activating mutations blocks mineralization and induces apoptosis in osteoblasts. J Cell Biol.

